# Risk factors for bronchiolitis obliterans in children with community-acquired pneumonia and analysis of CT findings and clinical manifestations of pneumonia after the diagnosis of bronchiolitis obliterans

**DOI:** 10.1080/07853890.2025.2578730

**Published:** 2025-10-30

**Authors:** Jiapu Hou, Ruiyang Sun, Xue Zhang, Wanyu Jia, Peng Li, Zhipeng Jin, Chunlan Song

**Affiliations:** Henan Children’s Hospital and Zhengzhou Children’s Hospital, Zhengzhou, Henan, China

**Keywords:** Bronchiolitis obliterans, community-acquired pneumonia, risk factors

## Abstract

**Objective:**

We explore the risk factors associated with the development of Bronchiolitis obliterans in children with community-acquired pneumonia to provide some basis for the early diagnosis of Bronchiolitis obliterans.

**Methods:**

We retrospectively analysed 83 children with community-acquired pneumonia who developed Bronchiolitis obliterans from January 2023 to October 2024 as the observation group, and 83 children with community-acquired pneumonia who did not develop Bronchiolitis obliterans for more than 1 year of follow-up as the control group. We performed a one-way logistic regression analysis of the clinical data of the two groups and further performed a multifactorial logistic regression analysis to determine the independent risk factors for the development of bronchiolitis obliterans in children with pneumonia. We analysed the ROC curves to determine the cut-off value of the indicator with the greatest diagnostic value.

**Results:**

The results of univariate and multivariate logistic regression analyses revealed that the independent risk factors for the development of bronchiolitis obliterans in children with pneumonia were age in months (OR = 0.982, *p* = 0.011), days of hospitalization (OR = 1.132, *p* = 0.043), dyspnoea (OR = 21.374, *p* < 0.001), pulmonary consolidation (OR = 5.267, *p* = 0.016) and endobronchitis (OR = 6.421, *p* = 0.002). The areas under the ROC curves were 0.700, 0.707, 0.711, 0.702 and 0.764 (*p* < 0.001), respectively. The critical values for months and days of hospitalization were 83 months and 10 days. In this study, 24 children in the observation group had bronchiectasis, 22 had bronchial stenosis, and 8 had pulmonary necrosis after the diagnosis of bronchiolitis obliterans.

**Conclusions:**

In children with community-acquired pneumonia who are less than 83 months of age, have been hospitalised for more than 10 days, and have dyspnoea, pulmonary consolidation, and endobronchitis, we should be vigilant for bronchiolitis obliterans to achieve early intervention and treatment.

## Introduction

Bronchiolitis obliterans (BO) is a rare and serious chronic obstructive pulmonary disease characterized by fibrosis involving the small conducting airways, which in turn leads to stenosis and complete obstruction of the airways [1,2]. Children with BO often present with symptoms of irreversible airway obstruction [2]. Infections, bone marrow transplants, connective tissue diseases, and side effects of certain medications such as amiodarone, certain chemotherapy drugs, etc. can lead to the development of BO [3,4]. It has been shown in previous literature that BO may be associated with autoimmune disorders, especially rheumatoid arthritis, systemic lupus erythematosus, Sjogren’s syndrome and less commonly with inflammatory bowel disease [5,6].

BO is categorized into three types: post-infectious BO (PIBO), post-lung transplantation BO, and post-bone marrow transplantation or hematopoietic stem cell transplantation BO [7]. In children, PIBO is the most common type of BO. Previous studies have shown that most of these infections are caused by adenovirus infections, followed by *Mycoplasma pneumoniae* infections [7,8]. With respect to disease progression from mild respiratory symptoms to persistent and severe symptoms of airway obstruction, the clinical manifestations of PIBO may vary. In previous relevant literature, the common risk factors for the development of BO in children with pneumonia are hypoxaemia, persistent wheezing, mechanical ventilation, length of hospital stay, higher levels of serum lactate dehydrogenase (LDH) and fever duration [9–12]. There is no effective treatment for PIBO in children, and clinical strategies regarding PIBO have limitations in reversing peribronchial fibrosis [13]. Early recognition and diagnosis of PIBO in children is therefore essential to reduce the burden of disease and improve the prognosis of children. However, the time lag between respiratory infection and symptom onset, the limitations of pulmonary function testing in children, and the non-specific symptoms of a proportion of children with PIBO pose obstacles to early diagnosis by clinicians. Therefore, in this study, we included the clinical data of 83 children with community-acquired pneumonia with legacy BO and 83 children without legacy BO. The objective of this study was to explore the risk factors associated with the development of Bronchiolitis obliterans in children with community-acquired pneumonia

## Materials and methods

### Study population

This is a case–control study in a retrospective study. In this study, we included 83 children with community-acquired pneumonia who developed BO at the Affiliated Henan Children’s Hospital and Zhengzhou Children’s Hospital from January 2023 to October 2024 as the observation group and another 83 children with community-acquired pneumonia of the same period who did not develop BO within more than one year of follow-up as the control group.

### Inclusion and exclusion criteria

The diagnostic criteria for BO are defined as follows [14,1]: Antecedent history: previous history of pneumonia or injury to the fine bronchi from other causes [2]; clinical manifestations: persistent or repeated wheezing or coughing, shortness of breath, dyspnoea, and exercise intolerance. Widespread wheezing and wet rales can be heard in both lungs and persist for more than 6 weeks, with a poor response to bronchodilators [3]. Adjunctive examination: chest CT revealed typical mosaic perfusion signs, bronchial dilatation, and bronchial wall thickening. Pulmonary function shows small airway obstructive ventilatory dysfunction or mixed ventilatory dysfunction, and bronchodilator tests are mostly negative. A diagnosis is made when the above conditions are met.

The diagnostic criteria for community-acquired pneumonia are defined as follows [15,1]: Clinical manifestations: new onset of fever, cough, sputum, wheezing, increased respiration, etc., or aggravation of preexisting respiratory symptoms with purulent sputum, with or without chest pain. Moist rales can be heard in the lungs [2]. Chest X-ray image showing lamellar or patchy infiltrative shadows or interstitial changes in the lungs, with or without pleural effusion. The diagnosis can be made if the above conditions are met.

The inclusion criteria for the observation group were as follows [1]: aged greater than 1 month and less than 18 years [2]; fulfilled the diagnostic criteria for BO, and the diagnosis of BO was first confirmed at our hospital; and [3] had a clear past history of pneumonia infection.

The control group inclusion criteria were as follows [1]: age greater than 1 month and less than 18 years and [2] diagnosis in accordance with the Community-Acquired Pneumonia in Children (2019 Edition) [3]. No BO occurred for more than 1 year of follow-up.

The exclusion criteria were as follows [1]: children with other serious diseases, such as connective tissue diseases, malignant tumors, and hematologic diseases [2]; children with a history of bone marrow transplantation or organ transplantation; and [3] children with incomplete clinical data and those for whom the prognosis of disease could not be determined.

### Data collection

**Figure 1.**
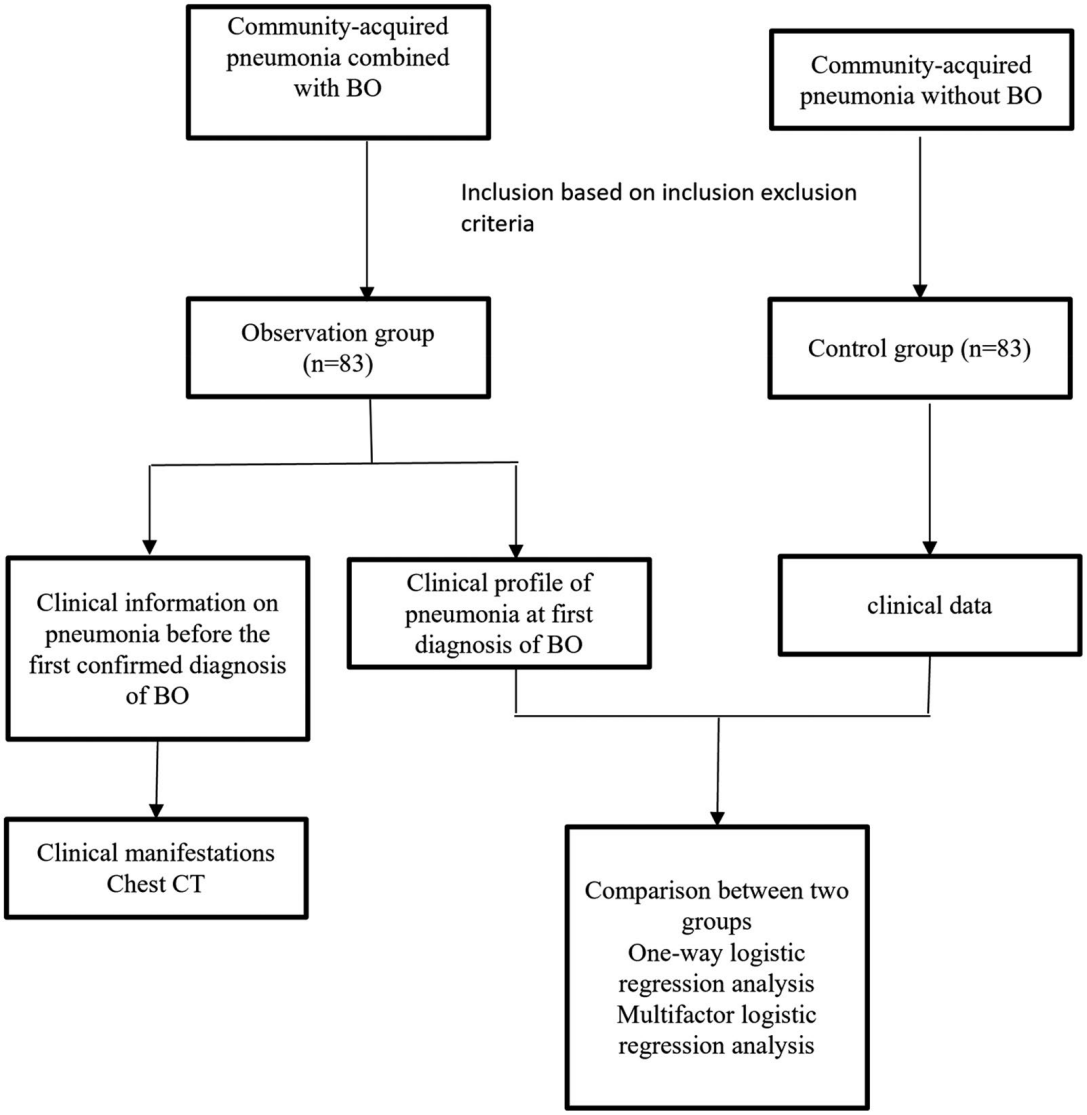
Flow chart.

We retrospectively collected clinical data on pneumonia before and at the time of the first diagnosis of BO in the children in the observation group and retrospectively collected relevant clinical data during the period of pneumonia in the children in the control group (Figure 1), including general information such as sex, age, and number of days of hospitalization; clinical manifestations such as days of fever, cough, wheezing, shortness of breath, and respiratory distress; chest CT and fibreoptic bronchoscopy; and laboratory examination results such as routine blood counts, biochemical indices, coagulation function, immune function, inflammatory indicators and other laboratory test results.

### Statistical analyses

We used SPSS software (version 21.0) for the statistical analysis. Quantitative information that conformed to a normal distribution is expressed as the mean ± standard deviation, and differences between groups were compared *via* independent samples *t*-tests. Quantitative information that did not conform to a normal distribution was expressed as the median P50 (P25, P75) and compared *via* the Mann–Whitney U-test. Qualitative information is expressed as a percentage (%). We performed one-way logistic regression analysis of statistically significant differences between the two groups. In one-way logistic regression analysis, variables with *p* < 0.05 were included in multifactorial logistic regression analysis to determine independent risk factors for the development of BO in children with community-acquired pneumonia. We used receiver operator characteristic (ROC) curves to assess the predictive value of age in months, length of hospitalization, dyspnoea, pulmonary consolidation, and endobronchitis on the risk of developing BO in children with community-acquired pneumonia. *p* < 0.05 indicated a statistically significant difference.

### Ethics approval and consent to participate

This study was approved by the Medical Ethics Committee of Henan Children’s Hospital and Zhengzhou Children’s Hospital (2024-067-001). Given the retrospective nature of the study, the Medical Ethics Committee of Henan Children’s Hospital and Zhengzhou Children’s Hospital has agreed to waive informed consent. The study adhered to the ethical standards of the Declaration of Helsinki.

## Results

### Comparison of the clinical data between the two groups of children

The age of the 83 children in the observation group was 34.00 (17.00, 81.00) months, the male to female ratio was 1.68:1, the number of days of hospitalization was 11.00 (7.00, 15.00) days, and the interval between pneumonia prior to the diagnosis of BO and the diagnosis of BO was 54 (25, 158) days. Information about the children in the control group is shown in Table 1. In the present study, both groups of children underwent chest CT and a total of 68 children underwent bronchoscopy.

**Table 1.** Comparison of baseline characteristics and clinical presentation between the two groups of children.

	Observation group (*n* = 83)	Control group (*n* = 83)	P
Sex (male)	62.70%	51.80%	0.158
Age in months	34.00 (17.00,81.00)	84.00 (40.00,108.00)	<0.001
Days of hospitalization	11.00 (7.00,15.00)	8.00 (7.00,9.00)	<0.001
Number of fever days	8.00 (4.00,11.50)	5.00 (1.00,7.00)	<0.001
Cough	94.00%	97.60%	0.247
Wheeze	37.30%	20.50%	0.017
Shortness of breath	54.20%	12.00%	<0.001
Dyspnoea	55.40%	13.30%	<0.001
Three depressions sign	36.10%	12.00%	<0.001

**Table 2.** Comparison of chest CT, bronchoscopy and laboratory tests between the two groups of children.

	Observation group (*n* = 83)	Control group (*n* = 83)	P
Pulmonary consolidation	75.40%	34.90%	<0.001
Pulmonary necrosis	7.80%	0.00%	0.010
Pulmonary atelectasis	12.30%	2.40%	0.017
Pulmonary embolism	3.10%	0.00%	0.105
Bronchiectasis	23.10%	0.00%	<0.001
Bronchial stenosis	18.50%	1.20%	<0.001
Bronchial wall thickening	6.20%	0.00%	0.022
Pleural effusion	33.80%	4.80%	<0.001
Pleural thickening	32.30%	9.60%	0.001
Number of bronchoscopies	1.00 (0.00,1.00)	0.00 (0.00,1.00)	<0.001
Endobronchitis	71.00%	18.10%	<0.001
Bronchial necrosis	4.80%	1.20%	0.418
Bronchial sputum embolism	14.50%	0.00%	0.001
White blood cell count (10^9/L)	10.76 ± 5.08	8.82 ± 3.48	0.013
Neutrophil count (10^9/L)	6.58 (3.56,10.03)	4.50 (2.94,6.98)	0.015
C-reactive protein (mg/L)	6.06 (0.50,22.24)	4.06 (0.50,13.41)	0.651
ESR (mm/h)	34.55 ± 24.31	30.83 ± 16.36	0.331
LDH (U/L)	356.00 (263.43,476.25)	300.50 (257.75,350.50)	0.007

Exegesis: Endobronchitis refers to the inflammation of the bronchial mucosa and bronchial lining, confirmed by bronchoscopy or histopathology.

There was no statistically significant difference in sex between the two groups of children. In the observation group, the age of the children was younger than that in the control group, the number of days of hospitalization and the number of days of fever were significantly greater than those in the control group, and the difference was statistically significant. The proportions of wheezing, shortness of breath, dyspnoea, and triple concave signs were significantly greater in the observation group than in the control group, and the difference was statistically significant. In terms of the chest CT manifestations of pulmonary consolidation, lung atelectasis, lung necrosis, bronchodilatation, bronchial stenosis, pleural thickening, and pleural effusion, the number of children in the observation group was significantly greater than that in the control group, and the difference was statistically significant. The number of bronchoscopies, cases of endobronchitis, and bronchial sputum embolisms in children in the observation group was significantly greater than that in the control group, and the difference was statistically significant. The white blood cell count, neutrophil count, and LDH level of the children in the observation group were significantly greater than those in the control group, and the difference was statistically significant. The differences between the two groups in terms of pulmonary embolism, bronchial necrosis, the erythrocyte sedimentation rate (ESR), and the C-reactive protein level were not statistically significant. (Tables 1,2)

In the observation group, the results of respiratory pathogenicity testing of nasopharyngeal swabs and sputum cultures showed 46 cases of viral infections, 53 cases of Mycoplasma pneumoniae infections, and 28 cases of bacterial infections. In the control group, the results of nasopharyngeal swabs and sputum cultures showed 27 cases of viral infections, 54 cases of Mycoplasma pneumoniae infections and 32 cases of bacterial infections. BAL was performed in 45 children in the observation group. Twenty-three children in the control group.

### Risk factor analysis and ROC curve for the occurrence of BO in children with community-acquired pneumonia

**Figure 2.**
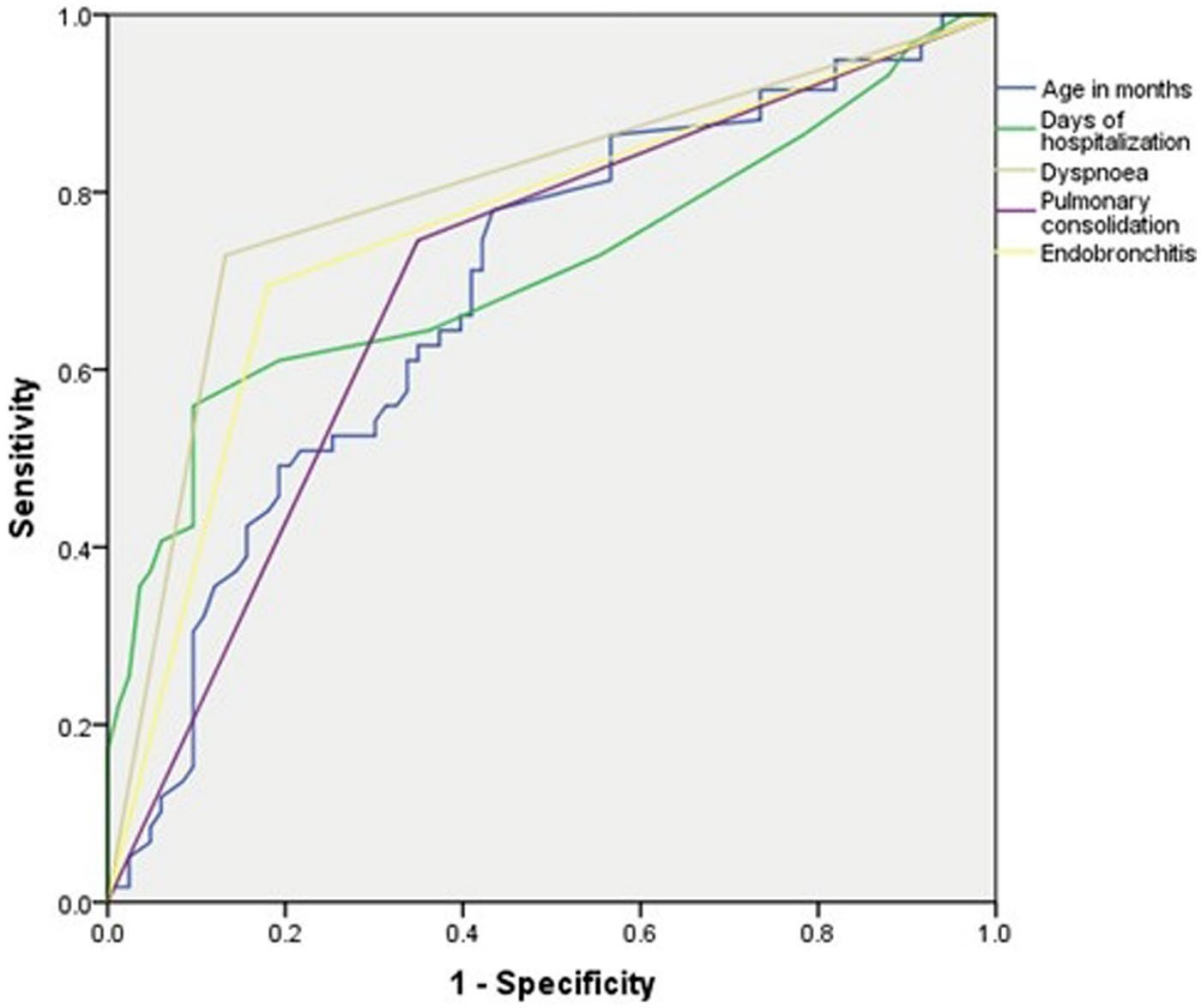
ROC curve.

Using one-way logistic regression analysis, we found a statistically significant effect of 15 risk factors on the occurrence of BO in children with community-acquired pneumonia. Among children with community-acquired pneumonia who developed BO, the younger the child initially diagnosed with community-acquired pneumonia, the greater the number of days of hospitalization and fever, the greater the levels of wheezing, shortness of breath, dyspnoea, three depression signs, pulmonary consolidation, pulmonary atelectasis, endobronchitis, bronchial stenosis, pleural effusion, pleural thickening, and LDH, and the greater the number of bronchoscopies, the greater the risk of developing PIBO. The results of multifactorial logistic regression analysis revealed that low monthly age, longer days of hospitalization, the presence of dyspnoea, pulmonary consolidation, and endobronchitis were independent risk factors for residual BO in children with community-acquired pneumonia. ROC curves were plotted for risk factors for the development of BO in children with community-acquired pneumonia (age in months, days of hospitalization, dyspnoea, pulmonary consolidation, and endobronchitis). The area under the ROC curve (AUC) was 0.700, 0.707, 0.711, 0.702 and 0.764 (*p* < 0.001), respectively. The cut-off values for age in months and days of hospitalization to predict the occurrence of BO in children with community-acquired pneumonia were 83 months (sensitivity 79.52%, specificity 56.63%) and 10 days (sensitivity 55.56%, specificity 90.36%), respectively (Tables 3, 4, Figure 2)

**Table 3.** Univariate and multivariate logistic regression analysis of risk factors for the development of BO in children with pneumonia.

Factors	Univariate analysis		Multivariate analysis	
	P	OR (95%CI)	P	OR (95%CI)
Age in months	<0.001	0.983 (0.975–0.991)	0.011	0.982 (0.968,0.996)
Days of hospitalization	<0.001	1.248 (1.134,1.373)	0.043	1.132 (1.004,1.277)
Dyspnoea	<0.001	8.138 (3.775,17.541)	<0.001	21.374 (6.037,75.667)
Pulmonary consolidation	<0.001	5.703 (2.769,11.746)	0.016	5.267 (1.364, 20.335)
Endobronchitis	<0.001	11.081 (5.064,24.250)	0.002	6.421 (1.964, 20.987)
Number of fever days	<0.001	1.194 (1.095,1.301)		
Wheeze	0.180	2.314 (1.156,4.634)		
Shortness of breath	<0.001	8.645 (3.926,19.037)		
Three depressions sign	<0.001	4.132 (1.860,9.181)		
Pulmonary atelectasis	0.032	5.684 (1.164,27.766)		
Bronchial stenosis	0.006	18.56 (2.345,146.991)		
Pleural effusion	<0.001	10.105 (3.270,31.226)		
Pleural thickening	0.001	4.474 (1.828,10.954)		
Number of bronchoscopies	<0.001	5.862 (2.999,11.457)		
White blood cell count (10^9/L)	0.010	1.115 (1.026,1.211)		
Neutrophil count (10^9/L)	0.011	1.136 (1.030,1.253)		
LDH (U/L)	0.002	1.005 (1.002,1.008)		

**Table 4.** ROC Curve analysis of risk factors for the development of BO in children with community-acquired pneumonia.

	AUC	P	Cut-off	Sensitivity (%)	Specificity (%)	Youden index
Age in months	0.700	<0.001	83.0	79.52	56.63	0.361
Days of hospitalization	0.707	<0.001	10.0	55.56	90.36	0.459
Dyspnoea	0.711	<0.001		55.42	86.75	0.422
Pulmonary consolidation	0.702	<0.001		75.38	65.06	0.404
Endobronchitis	0.764	<0.001		70.97	81.93	0.529

### .Clinical manifestations and chest CT manifestations of children in the observation group after diagnosis of BO

**Figure 3.**
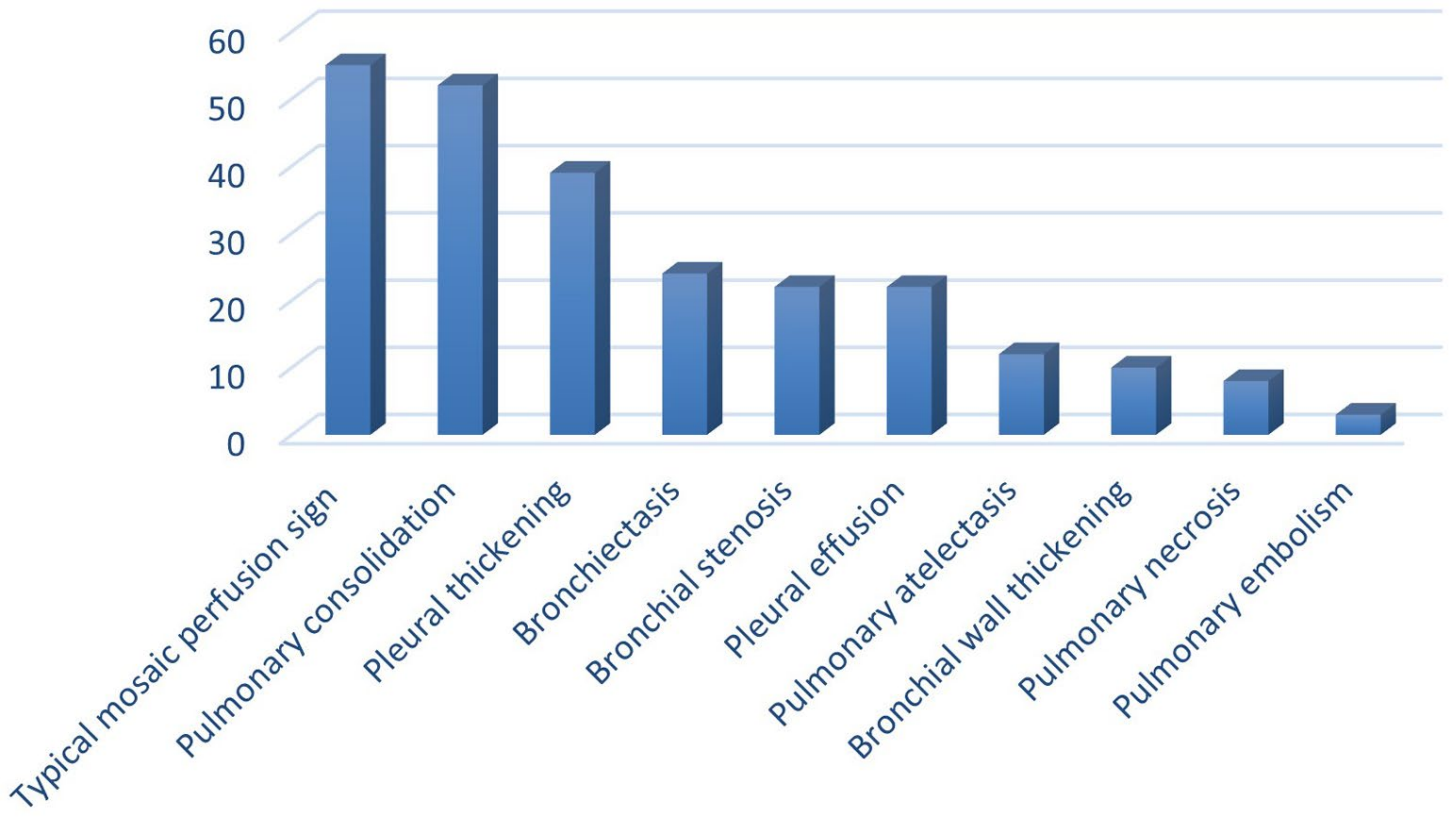
Chest CT findings in children after diagnosis of BO.

**Figure 4.**
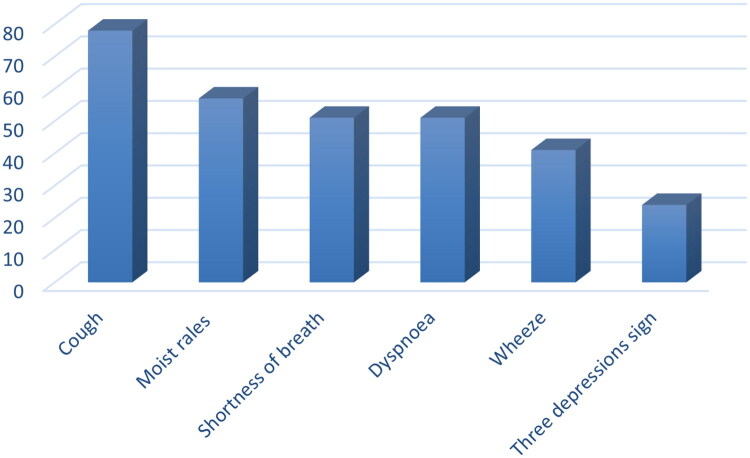
Clinical manifestations of children after diagnosis of BO.

After the diagnosis of BO was made in 83 children in the observation group, more than 60% of the children presented with cough, shortness of breath, dyspnoea, and wet rales in the lungs, and approximately 50% presented wheezing symptoms. On chest CT, 55 patients had typical mosaic perfusion signs, 52 had solid lesions in the lungs, 24 had residual bronchodilatation, 39 had pleural thickening, and 10 had thickening of the bronchial wall. (Figures 3, 4)

## Discussion

BO, also known as constrictive bronchiectasis, is a rare chronic obstructive respiratory disease of the small airways. The main mechanism is damage to the airway epithelium and the immediately following proliferation of fibroblasts, which leads to the narrowing and occlusion of the fine bronchial tubes of the airways [10]. Currently, there are fewer domestic and international studies related to the analysis of risk factors for the development of BO in children with pneumonia, and the diagnosis of BO in children with community-acquired pneumonia is not yet adequate [10,16]. In this study, we identified five independent risk factors for the occurrence of BO in children with community-acquired pneumonia *via* multifactorial logistic regression analysis and described the clinical and CT manifestations at the time of the first diagnosis of BO in the hope of providing some basis for the early diagnosis of children with PIBO.

In our study, the age of the child in months was an independent risk factor for BO after pneumonia. The younger the child is in months, the greater the risk of BO. Children with pneumonia who are younger than 83 months of age are more likely to have BO. We hypothesize that this may be related to the immunocompromised nature of the child at a young age [17]. In the study by Die Liu et al. it was stated that children who developed PIBO were younger than those in the control group were [18]. It has also been shown in this literature that younger children and males have a greater risk of PIBO than older children do [18]. This finding is consistent with our findings. Therefore, special attention should be given to young boys with community-acquired pneumonia. Length of hospitalization was the second independent risk factor for the development of BO in children with community-acquired pneumonia. Children who were hospitalized for more than 10 days in this study were at greater risk of BO. An association between the number of days of hospitalization after infection and the development of PIBO has been reported in previous studies, with children who developed PIBO having longer postinfection hospitalizations [18,19]. This conclusion is consistent with the results of our study. The length of hospitalization reflects the severity of the child’s pneumonia. Persistent infection and the inflammatory response during prolonged hospitalization may lead to abnormal damage repair in lung tissue [20]. Especially in the bronchial area, prolonged stimulation by inflammatory factors leads to pulmonary fibrosis and airway narrowing, promoting BO [20]. Moreover, we hypothesized that prolonged hospitalization may mean that the children did not receive effective anti-infective treatment in the early stages and that the inflammation and immune response were not effectively controlled, further damaging the bronchial tubes and alveoli.

In this study, dyspnoea was the third independent risk factor for the development of BO in children with pneumonia. In the present study, the proportion of children with dyspnoea in the observation group was significantly greater than that in the control group, indicating that children with pneumonia presenting with dyspnoea are at a greater risk of developing BO. Independent risk factors for BO were fever duration, dyspnoea, and invasive mechanical ventilation in a study by Linping Zhong et al. which is consistent with the results of this study [19]. Several previous studies have shown that hypoxemia is a risk factor for BO in children with pneumonia [10,18,21]. Children with severe pneumonia are prone to respiratory distress, hypoxemia and other clinical manifestations. If hypoxia is not corrected in time, it can easily lead to further aggravation of airway inflammation and lung injury and even irreversible lung injury. Airway injury and inflammation further promote fibroblast overgrowth, abnormal tissue repair and remodelling of the fine bronchial wall, leading to the development of BO [7,20].

Fiberoptic bronchoscopy revealed the presence of endobronchitis in a greater proportion of children in the observation group than in the control group. Multifactorial logistic regression analysis revealed that endobronchitis was an independent risk factor for the development of BO in children with pneumonia. Some relevant studies have shown that the main mechanism of BO occurrence is related to damage to the airway epithelium and the subsequent proliferation of fibroblasts [22,23]. Infection leads to damage and shedding of airway epithelial cells, causing an inflammatory response that manifests itself on bronchoscopy as endobronchitis and promotes the development of BO. Therefore, children with endobronchitis are more likely to develop BO. In this study, pulmonary consolidation was the last independent risk factor for the development of BO in children with community-acquired pneumonia. Solid lesions in the lobes of the lungs are imaging manifestations of lung lesions. It reflects the presence of extensive areas of the inflammatory response and lesions in the lungs [20]. We hypothesized that more aggressive interventions during the acute phase of pneumonia in children with pulmonary consolidation may reduce the inflammatory response and lower the risk of BO.

In previous relevant literature, the common risk factors for the development of BO in children with pneumonia are hypoxaemia, persistent wheezing, mechanical ventilation, length of hospital stay, higher levels of LDH and fever duration [9–12]. In addition, this study revealed that the number of days of fever, pulmonary atelectasis, pleural effusion, leukocyte count, neutrophil count, and lactate dehydrogenase levels of the children in the observation group were greater than those in the control group. One-way logistic regression analysis revealed a statistically significant effect of these factors on the occurrence of PIBO (*p* < 0.05). Fever is an immune response of the body to infection. The number of fever days is closely related to the intensity of the inflammatory response. Previous studies have shown that persistent fever in children with pneumonia tends to cause damage to the epithelial cells of the bronchial mucosa, which in turn promotes the infiltration of inflammatory cells and increases the activity of fibroblasts, thus inducing abnormal fibrosis and obstruction of the fine bronchial tubes [20]. Moreover, prolonged fever may also disrupt the body’s immune function, resulting in the body’s inability to remove infectious agents in a timely and effective manner, further exacerbating bronchial damage and remodelling and ultimately promoting the development of BO.

Pulmonary atelectasis is an intrapulmonary complication in children with community-acquired pneumonia that causes impaired ventilation and localized hypoxia in lung tissue [24]. Moreover, the occurrence of pulmonary atelectasis can exacerbate the inflammatory response of the bronchial tubes, which in turn leads to bronchial fibrosis and occlusion, causing BO [20]. Some studies have proposed that pleural effusion is a risk factor associated with the development of PIBO [13]. Some studies have concluded that the development of pleural effusion in children with pneumonia is associated with a more severe clinical course and a more severe form of pneumonia [25]. We therefore hypothesize that pleural effusion occurs, in part, as a reflection of a more intense inflammatory response that promotes BO. In previous relevant studies, elevated serum LDH levels were found to be an independent risk factor for the development of BO in children with severe *Mycoplasma pneumoniae* pneumonia, and serum LDH levels can reflect the extent of organ damage, especially in lung tissue [11,12]. We hypothesize that severe lung injury may induce excessive aberrant repair, which may play a role in the pathogenesis of BO. In addition, Tie-Hu Liu et al. reported that elevated LDH not only is a marker of lung tissue damage but also represents a more severe inflammatory response in the lungs, as well as fibrosis and occlusion of fine bronchioles, which may ensue [20]. Elevated white blood cell and neutrophil counts also reflect a strong inflammatory response in the body, which is capable of causing BO.

The characteristic CT manifestations of PIBO in children are the typical mosaic perfusion sign, thickening of the bronchial wall, bronchiectasis, and pulmonary atelectasis [26]. In our study, 55 children with BO in the observation group had typical mosaic perfusion manifestations. This result is consistent with the results of the current study. Moreover, 24 children in this study were diagnosed with BO combined with bronchiectasis, 22 with bronchial stenosis, and 8 with manifestations of pulmonary necrosis. It is suggested that in children with severe pneumonia, bronchiectasis, bronchial stenosis, and pulmonary necrosis may be left behind along with the occurrence of BO, which may aggravate the burden on the child. More long-term follow-up is needed for these children.

This study has several limitations. First, the sample size was small due to the rarity of children with BO, and the enrolled children were from a single center. Also insufficient sample size may affect model stability. The results need to be further validated with larger samples and multicenter studies. Second, this paper does not cover all the observations related to BO and needs further refinement.

## Conclusion

In conclusion, children with community-acquired pneumonia who are younger in months, have a long hospital stay, and have dyspnoea, pulmonary consolidation, and endobronchitis should be highly vigilant in the development of PIBO to achieve early intervention and recognition to avoid the occurrence of irreversible airway obstruction and thus improve the quality of life of these children.

## Acknowledgements

Not applicable.

## Data Availability

The datasets used and/or analyzed during the current study are available from the corresponding author on reasonable request.

## References

[1] Colom AJ, Teper AM. Post-infectious bronchiolitis obliterans. Pediatr Pulmonol. 2019;54(2):212–219. doi:10.1002/ppul.24221. 30548423

[2] Flanagan F, Casey A, Reyes-Múgica M, et al. Post-infectious bronchiolitis obliterans in children. Paediatr Respir Rev. 2022;42:69–78. doi:10.1016/j.prrv.2022.01.007. 35562287

[3] Jerkic S-P, Brinkmann F, Calder A, et al. Postinfectious Bronchiolitis Obliterans in Children: diagnostic Workup and Therapeutic Options: a Workshop Report. Can Respir J. 2020;2020:5852827. doi:10.1155/2020/5852827. 32076469 PMC7013295

[4] Spagnolo P, Bonniaud P, Rossi G, et al. Drug-induced interstitial lung disease. Eur Respir J. 2022;60(4):2102776. doi:10.1183/13993003.02776-2021. 35332071

[5] Ryu JH, Azadeh N, Samhouri B, et al. Recent advances in the understanding of bronchiolitis in adults. F1000Res. 2020;9:568. doi:10.12688/f1000research.21778.1. PMC7281671 32551095

[6] Swaminathan AC, Carney JM, Tailor TD, et al. Overview and Challenges of Bronchiolar Disorders. Ann Am Thorac Soc. 2020;17(3):253–263. doi:10.1513/AnnalsATS.201907-569CME. 31860801

[7] Kavaliunaite E, Aurora P. Diagnosing and managing bronchiolitis obliterans in children. Expert Rev Respir Med. 2019;13(5):481–488. doi:10.1080/17476348.2019.1586537. 30798629

[8] Yu X, Ma Y, Gao Y, et al. Epidemiology of Adenovirus Pneumonia and Risk Factors for Bronchiolitis Obliterans in Children During an Outbreak in Jilin, China. Front Pediatr. 2021;9:722885. doi:10.3389/fped.2021.722885. 34650942 PMC8506152

[9] Yao M-M, Gao T-J, Zhao M, et al. Risk factors for bronchiolitis obliterans complicating adenovirus pneumonia in children: a meta-analysis. Front Pediatr. 2024;12:1361850. doi:10.3389/fped.2024.1361850. 39149537 PMC11324480

[10] Huang K, Liu J, Lv W, et al. Analysis of Risk Factors of Bronchiolitis Obliterans in Children with Mycoplasma pneumoniae Bronchiolitis. Comput Math Methods Med. 2022;2022:9371406. doi:10.1155/2022/9371406. 35242215 PMC8886696

[11] Lee E, Young Lee Y. Risk factors for the development of post-infectious bronchiolitis obliterans after Mycoplasma pneumoniae pneumonia in the era of increasing macrolide resistance. Respir Med. 2020;175:106209. doi: 10.1016/j.rmed.2020.106209. 33186845

[12] Zheng HQ, Ma YC, Chen YQ, et al. Clinical analysis and risk factors of bronchiolitis obliterans after mycoplasma pneumoniae pneumonia. Infect Drug Resist. 2022;15:4101–4108. doi:10.2147/IDR.S372940. 35924019 PMC9343175

[13] Lee E, Park S, Kim K, et al. Risk factors for the development of post-infectious bronchiolitis obliterans in children: a systematic review and meta-analysis. Pathogens. 2022;11(11):1268. doi:10.3390/pathogens11111268. PMC9696236 36365019

[14] Diseases TSGoR. Consensus on the diagnosis and treatment of bronchiolitis obliterans in children. Chinese Journal of Pediatrics. 2012;50(10):743–745. 23302561

[15] China NHCotPsRo, Medicine SAoTC. Guideline for diagnosis and treatment of community-acquired pneumonia in Children (2019 version). Chinese Journal of Clinical Infectious Diseases. 2019;12(1):6–13.

[16] Yuan J, Wei M, Chen M, et al. Risk factors for the development of bronchiolitis obliterans in children after suffering from adenovirus pneumonia. Front Pediatr. 2023;11:1335543. doi:10.3389/fped.2023.1335543. 38269287 PMC10806191

[17] Wen S, Xu M, Jin W, et al. Risk factors and prediction models for bronchiolitis obliterans after severe adenoviral pneumonia. Eur J Pediatr. 2024;183(3):1315–1323. doi:10.1007/s00431-023-05379-1. 38117354

[18] Liu D, Liu J, Zhang L, et al. Risk Factors for Post-infectious Bronchiolitis Obliterans in Children: A Systematic Review and Meta-Analysis. Front Pediatr. 2022;10:881908. doi:10.3389/fped.2022.881908. 35757133 PMC9218415

[19] Zhong L, Lin J, Dai J. Risk factors for the development of bronchiolitis obliterans in children with severe adenovirus pneumonia: A retrospective study with dose-response analysis. J Med Virol. 2020;92(12):3093–3099. doi:10.1002/jmv.25703. 32068263

[20] Liu TH, Liu XX, Tang Y, et al. [Construction of a risk prediction model for bronchiolitis obliterans in children with refractory Mycoplasma pneumoniae pneumonia]. Zhongguo Dang Dai Er Ke Za Zhi. 2024;26(9):946–953. doi:10.7499/j.issn.1008-8830.2402008. 39267510 PMC11404459

[21] Wu P-Q, Li X, Jiang W-H, et al. Hypoxemia is an independent predictor of bronchiolitis obliterans following respiratory adenoviral infection in children. Springerplus. 2016;5(1):1622. doi:10.1186/s40064-016-3237-7. 27722041 PMC5030207

[22] Zhao C, Liu J, Yang H, et al. Mycoplasma pneumoniae-associated bronchiolitis obliterans following acute bronchiolitis. Sci Rep. 2017;7(1):8478. doi:10.1038/s41598-017-08861-7. 28814783 PMC5559585

[23] Mazenq J, Dubus JC, Chanez P, et al. Post viral bronchiolitis obliterans in children: A rare and potentially devastating disease. Paediatr Respir Rev. 2024;52:58–65. doi:10.1016/j.prrv.2024.04.003. 39214823

[24] Miyashita N, Kawai Y, Inamura N, et al. Setting a standard for the initiation of steroid therapy in refractory or severe Mycoplasma pneumoniae pneumonia in adolescents and adults. J Infect Chemother. 2015;21(3):153–160. doi:10.1016/j.jiac.2014.10.008. 25533771

[25] Kim SH, Lee E, Song ES, et al. Clinical significance of pleural effusion in mycoplasma pneumoniae pneumonia in children. Pathogens. 2021;10(9):1075. doi:10.3390/pathogens10091075. 34578108 PMC8469935

[26] Kim J, Kim M-J, Sol IS, et al. Quantitative CT and pulmonary function in children with post-infectious bronchiolitis obliterans. PLoS One. 2019;14(4):e0214647. doi:10.1371/journal.pone.0214647. 30934017 PMC6443232

